# The p.P56S mutation in the *VAPB* gene is not due to a single founder: the first European case

**DOI:** 10.1111/j.1399-0004.2009.01319.x

**Published:** 2010-03

**Authors:** AD Funke, M Esser, A Krüttgen, J Weis, M Mitne-Neto, M Lazar, AL Nishimura, AD Sperfeld, P Trillenberg, J Senderek, M Krasnianski, M Zatz, S Zierz, M Deschauer

**Affiliations:** a Department of Neurology, Martin-Luther-Universität Halle (Saale)Germany; b Institute of Neuropathology, Rheinisch-Westfälische Technische Universität AachenGermany; c Centro de Estudos do Genom Humano, Instituto de Biociencias, Departamento de Biologia, Universidade de Sao PauloBrazil; d Department of Neurology, Universität UlmGermany; e Department of Neurology, Universitätsklinikum Schleswig-Holstein, Campus LübeckGermany

*To the Editor*:

A dominant missense mutation p.P56S in the *vesicle associated membrane protein associated protein B* (*VAPB*) gene was described in eight Brazilian families of Portuguese descent showing a wide spectrum of motor neuron diseases (MNDs) including spinal muscular atrophy (SMA) and familial amyotrophic lateral sclerosis (ALS) (ALS8) (1, 2). Haplotype analysis indicated a common ancestor with a founding event 23 generations previously, when this ancestor was still living in Portugal (3). We report the first identification of the p.P56S mutation in the *VAPB* gene in a non-Brazilian patient.

**Fig. 1:**
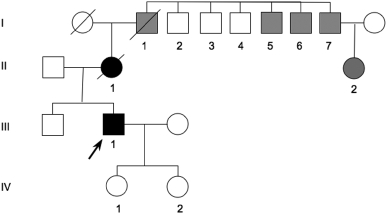
Family tree. Family members who are deemed affected based upon family history are marked gray. It is not known if family members I-2, I-3, I-4, I-5, I-6, I-7, and II-2 are still alive.

A 43-year-old man (III-1) showed slowly progressive muscular weakness for 2 years and a family history of autosomal dominant neuromuscular disease through at least three generations (Fig 1). The patient's mother (II-1) suffered from slowly progressive muscular weakness over 30 years. No pyramidal tract signs had been observed. She was wheelchair bound 20 years after onset and died at the age of 67. The maternal grandfather (I-1) had a history of progressive muscular weakness, had died aged 57 and had six siblings. Three (I-5, I-6, I-7) suffered from muscular weakness. One cousin of the patient's mother (II-2) was diagnosed with SMA. There was no family record of Portuguese or Brazilian ancestors in at least four previous generations. All family members originated from northern Germany.

The index patient (III-1) showed paresis of the hip flexors and extensors (Medical Research Council (MRC) grade 4/5) and fasciculations in the proximal muscles of arms, legs on both sides. Deep tendon reflexes were normal except for absent Achilles tendon reflexes. There were no pyramidal tract signs. Needle electromyography showed fasciculations and signs of chronic denervation. Nerve conduction studies of tibial nerves revealed slightly reduced amplitudes on the left. Motor evoked potentials in both tibialis anterior muscles after magnetic stimulation of the motor cortex and the lumbar roots were normal.

Genomic DNA of the index case was extracted from peripheral blood and amplified using primer pairs flanking all exons and exon/intron boundaries of the *VAPB* gene. Amplicons were purified and sequenced directly on an ABI PRISM 310 Genetic Analyzer (PE, Applied Biosystems). The p.P56S mutation was screened in 100 German healthy controls. Haplotype analysis was performed using microsatellite markers D20S100, D20S171 and D20S173 from the ABI Prism Linkage Mapping Set kit version 2 (Applied Biosystems, Foster City, CA) as reported previously (3). The forward 5′AAGACAAGCAAAACTAAAGAACTGC3′and reverse 5′TTCCCATTACCGGTTATCCA-3′ primers were used to amplify part of 3′ UTR sequence of the tubulin beta 1 (TUBB1) gene. A polymorphism in this region was used as intrafamilial marker. Polymerase chain reaction products were analyzed using a MegaBace 1000 DNA Sequencer (Amersham Bioscience, Little Chalfont, UK). DNA from the index patient was compared to three affected individuals from three different kindreds of the Brazilian families.

Sequencing revealed a heterozygous p.P56S point mutation in exon 2 of the *VAPB* gene. This mutation was not present in 200 German control chromosomes. Haplotype analysis revealed that this patient had a different haplotype compared to the Brazilian families (Table 1).

**Table 1:** Haplotype comparison of the German index patient and the patients from three different Brazilian families with the p.P56S *vesicle associated membrane protein associated protein B (VAPB)* gene mutation^a^

			Brazilian families		
Position (Mb)	Marker	German index patient	1	2	3
53.74	D20S100	222 232	224 **230**	226 **230**	232 **230**
57.0	TUBB1	G G	G **C**	G **C**	G **C**
57.24	D20S171	142 142	142 **144**	144 **144**	140 **144**
58.31	D20S173	176 178	178 **178**	176 **178**	178 **178**

a The haplotype given in boldface signifies the ancestral allele in the Brazilian families.

The phenotype of our index case and his mother represented late onset SMA as observed in one-third of the Brazilian patients carrying the p.P56S *VAPB* mutation (1). Although we cannot entirely exclude the possibility of Brazilian or Portuguese ancestors, haplotype analysis showed that our patient's mutation is not due to the same founder as in the reported Brazilian patients. Therefore, we assume that the p.P56S mutation happened in at least two independent events. Several studies failed to identify *VAPB* mutations in cohorts of patients with ALS (4–6)]. Mutations in the *VAPB* gene seem to be rare in familial MNDs. However, our case report demonstrates that the p.P56S mutation can be observed outside Brazil, and should be considered as a rare differential diagnosis in familial MNDs.
